# In Vitro Susceptibility Testing of Clinical Isolates of *Chlamydia trachomatis*

**DOI:** 10.1155/S1064744993000109

**Published:** 1993

**Authors:** Mark G. Martens, Sebastian Faro, Maurizio Maccato, Gerald Riddle, Hunter Hammill, Y. Wang

**Affiliations:** 1 Section of Infectious Diseases, Department of Obstetrics and Gynecology Baylor College of Medicine Houston TX USA; 2 Division of Infectious Diseases, Department of Obstetrics and Gynecology The University of Texas Medical Branch 3.108 Old Children's Hospital Galveston TX 77550 USA

## Abstract

Penicillin class antibiotics have demonstrated varying degrees of in vivo and in vitro success when tested against *Chlamydia trachomatis*. The activity of ampicillin-sulbactam, an agent commonly utilized in the treatment of pelvic infections, was tested to ascertain if any antichlamydial activity is present. Up to six endocervical isolates of *C. trachomatis* were tested against each of five antibiotics including doxycycline, erythromycin, clindamycin, ampicillin/sulbactam, and sulbactam alone. McCoy cell monolayers were inoculated with high inclusion counts of 10,000–30,000 inclusion-forming units (IFU) per coverslip, and exposed to each antibiotic. Up to nine subsequent antibiotic free culture passes were performed to assess the viability of abnormal inclusions. Doxycycline, erythromycin, and clindamycin achieved 100% eradication of inclusions at concentrations of 4.0, 2.0, and 1.0 µg/mL. Exposure to ampicillin/sulbactam resulted in a greater than 99% reduction in the inclusion count at 32.0 µg/mL, while sulbactam by itself demonstrated considerably less activity. Abnormal inclusions were noted only in the ampicillin/sulbactam exposed cells, and these, plus all inclusions remaining following sublethal exposure to the other antibiotics, resulted in regrowth to control levels in subsequent passes. Doxycycline and erythromycin demonstrated excellent activity. Clindamycin and ampicillin/sulbactam also significantly reduced inclusion formation, and therefore may provide adequate *C. trachomatis* coverage in patients receiving these antibiotics for pelvic infections.


Infectious Diseases in Obstetrics and Gynecology 1:40–45 (1993)
© 1993 Wiley-Liss, Inc.

### In Vitro Susceptibility Testing of Clinical Isolates of *Chlamydia trachomatis*

**Mark G. Martens, Sebastian Faro, Maurizio Maccato, Gerald Riddle, Hunter Hammill, and Y. Wang**

*Section of Infectious Diseases, Department of Obstetrics and Gynecology, Baylor College of Medicine, Houston, TX*

Abstract

**Penicillin class antibiotics have demonstrated varying degrees of in vivo and in vitro success when tested against *Chlamydia trachomatis*. The activity of ampicillin-sulbactam, an agent commonly utilized in the treatment of pelvic infections, was tested to ascertain if any antichlamydial activity is present. Up to six endocervical isolates of *C. trachomatis* were tested against each of five antibiotics including doxycycline, erythromycin, clindamycin, ampicillin/sulbactam, and sulbactam alone. McCoy cell monolayers were inoculated with high inclusion counts of 10,000–30,000 inclusion-forming units (IFU) per coverslip, and exposed to each antibiotic. Up to nine subsequent antibiotic free culture passes were performed to assess the viability of abnormal inclusions. Doxycycline, erythromycin, and clindamycin achieved 100% eradication of inclusions at concentrations of 4.0, 2.0, and 1.0 μg/mL. Exposure to ampicillin/sulbactam resulted in a greater than 99% reduction in the inclusion count at 32.0 μg/mL, while sulbactam by itself demonstrated considerably less activity. Abnormal inclusions were noted only in the ampicillin/sulbactam exposed cells, and these, plus all inclusions remaining following sublethal exposure to the other antibiotics, resulted in regrowth to control levels in subsequent passes. Doxycycline and erythromycin demonstrated excellent activity. Clindamycin and ampicillin/sulbactam also significantly reduced inclusion formation, and therefore may provide adequate *C. trachomatis* coverage in patients receiving these antibiotics for pelvic infections.**
© 1993 Wiley-Liss, Inc.

Key words

*Chlamydia trachomatis,* ampicillin-sulbactam, penicillins

*Chlamydia trachomatis* is one of the most prevalent sexually transmitted organisms of the female genital tract.[1] Infection may result in serious sequelae such as infertility, ectopic pregnancy, and pelvic inflammatory disease. Treatment regimens usually consist of tetracycline or erythromycin; however, sulfa drugs and clindamycin have demonstrated good activity.[2,3]

Penicillin-based antibiotics have demonstrated efficacy in some in vitro studies,[4,5] and in fact have recently been recommended as an alternative therapy to erythromycin in the treatment of chlamydial infection in pregnancy.[6] However, due to the elongated life cycle of *C. trachomatis,* single dose therapy with penicillin G, tetracycline, or erythromycin does not cure cases of urethritis caused by *C. trachomatis.*[6]

Penicillins have also been noted to produce abnormal inclusions, which may revert to normal at a later time.[7] This observation may have possibly discouraged the study of newer broad spectrum penicillins for treatment of *C. trachomatis* infections despite early, although limited, clinical success. Penicillins have been reported to eradicate *C.*

Address correspondence/reprint requests to M.G. Martens, Division of Infectious Diseases, Department of Obstetrics and Gynecology, The University of Texas Medical Branch, 3.108 Old Children's Hospital, Galveston, TX 77550.

Received January 15, 1993
Accepted January 21, 1993

*trachomatis* in eight of ten women treated with a 21-day course of ampicillin for cervical and/or urethral infection.[8] Bowie et al. reported the microbiological cure in six of six men with urethritis when treated with a 10-day regimen of amoxicillin.[9] Martin et al. reported the microbiological cure of *C. trachomatis* infection in seven patients treated with ticarcillin/clavulanic acid, whereas three patients treated with cephalosporins remained culture positive after treatment.[5] These results correlated well with their in vitro susceptibility results of 19 isolates of *C. trachomatis* against broad spectrum semi-synthetic penicillin and cephalosporins. Ticarcillin, mezlocillin, and piperacillin exhibited good in vitro susceptibility results in 19 isolates of *C. trachomatis,* while cefoxitin, cefotaxime, and cefamandole exhibited poor in vitro activity.

Also, despite excellent in vitro activity, treatment failures with the current drugs of choice, tetracycline and erythromycin, have also been reported.[6–10] Similarly, in vitro resistance of erythromycin has been reported, but is rare.[11]

Several investigators have attempted to predict clinical efficacy of antibiotic regimens by performing in vitro susceptibility testing. However, because of the variety of in vitro susceptibility testing techniques that have been utilized, clinical correlations do not always agree.[2,4,7,8,12–17]

The present investigation provides additional information concerning the susceptibility pattern for various antibiotics, including ampicillin/sulbactam, which may be clinically useful in the treatment of chlamydial infections.

### SUBJECTS AND METHODS

### Source of Isolates

Endocervical specimens were collected from two patient populations: 1) nonpregnant adolescents attending a teen health clinic; and 2) postpartum patients who were evaluated for endometritis.

### Processing of Specimens

A Dacron swab was inserted in the endocervix after the portion of the cervix had been cleansed with a cotton-tipped applicator. The swab was rotated several times to collect cellular material and was then placed in *Chlamydia* transport medium. The transport tube was refrigerated (2–8°C) and kept on ice until delivery to the laboratory (within 24 hours).

Upon receipt in the laboratory, the transport tubes were vortexed for 30 seconds to remove cellular material from the fiber matrix. Excess moisture was removed from the swab by pressing it against the side of the tube as it was withdrawn, and the swab was then discarded. The tubes were centrifuged at 200*g* for 5 minutes to remove debris. The supernatant (0.3 mL) was used to inoculate McCoy cell monolayers grown on coverslips contained in 1-dram vials after the overlay medium had been removed. All transport tubes were then frozen at −70°C. The inoculated vials were centrifuged for 1 hour at 3,000g at 35°C for 1 hour. Fresh isolation medium was added, and the vials were incubated for an additional 48–72 hours before staining. The *Chlamydia* transport media, McCoy cell monolayers, and isolation medium were obtained from Bartels Immunodiagnostics (Bellevue, WA). The McCoy cell monolayers were stained with Jones' iodine or with *Chlamydia*-specific fluorescent antibody (FA) reagent (Ortho Diagnostic, Raritan, NJ).

### Susceptibility Testing

Stock solutions (100× highest concentration tested) were prepared in minimal essential medium (MEM). Aliquots were frozen at −70°C for no longer than 30 days before use. The concentrations (μg/mL) of the antibiotics tested were as follows: clindamycin 0.25, 0.50, and 1.0; doxycycline 4.0, 8.0, and 16.0; and sulbactam 3, 6, and 12. Slightly higher concentrations were utilized due to the high *Chlamydia* inoculum used. Ampicillin/sulbactam (Roerig, Groton, CT), were tested at a 2:1 ratio of ampicillin (6, 12, and 24 μg/mL) to sulbactam. The earlier penicillins have been tested extensively and therefore ampicillin was not included in this investigation.[2,4,5]

Frozen transport media was thawed and used to inoculate five vials of McCoy cells. After 48–72 hours, McCoy cell vials were prepared for passage by first centrifuging at 3,000g for 30 minutes to reassociate elementary bodies with the monolayer. The overlay medium was then decanted and replaced with 0.4 mL 2 sucrose phosphate medium (2SP). The McCoy cell vials were frozen twice at −70°C, thawed, and sonicated in an ultrasonic bath for 2 minutes. The sonicated suspensions were passed through a sterile 1.2-μm syringe tip filter to

**TABLE 1. Activity of antibiotics vs. *Chlamydia trachomatis***

| Antibiotic | No. of isolates | Concentration (μg/mL)[a] | | | | | | | |
|---|---|---|---|---|---|---|---|---|---|
| | | 0.25 | 0.5 | 1.0 | 2.0 | 4.0 | 8.0 | 16.0 | 32.0 |
| Sulbactam[b] | 5 | — | — | — | — | 0 | 64[c] | 68 | — |
| Ampicillin/sulbactam | 5 | — | — | — | — | — | 95 | 95 | 99 |
| Clindamycin | 6 | 99 | 99 | 100 | — | — | — | — | — |
| Doxycycline | 5 | — | — | — | — | 100 | 100 | 100 | — |
| Erythromycin | 5 | — | — | — | 100 | 100 | 100 | — | — |

[a]—, not tested.
[b]Concentrations utilized for sulbactam: 3, 6, and 12 μg/mL.
[c]Percentage of inclusions inhibited at each concentration of antibiotic.

remove cell debris. A 0.1 mL portion of the suspension was used to inoculate new vials. At least three passes were performed using MEM overlay media with antibiotics. An additional three passes were performed using MEM overlay media without antibiotics and with 5% heat inactivated fetal calf serum. McCoy cells were also maintained in MEM without antibiotics for 3 days prior to inoculation. An inoculum containing 10,000–30,000 IFUs per coverslip was obtained with this method.

A 0.1-mL portion of the final inoculum was added to each of four McCoy cell monolayers at each antibiotic concentration and centrifuged for 1 hour at 3,000g, 30°C. The monolayers were incubated for 1 hour at 35°C. Feeding MEM containing the desired concentrations of antibiotics tested was then added to the monolayer. The monolayers were incubated for 48 hours. Two of the monolayers were stained, and two were used for a passage into four new vials. Between the first and second passes, monolayers were washed with antibiotic-free MEM to remove residual antibiotic. For passage, 2 mL of 2SP media were added per vial. The cell layer was then disrupted with a Pasteur pipette, frozen at −70°C, thawed, refrozen, and sonicated. Additional passes were performed for each isolate until regrowth of initial inoculum size or no growth occurred up to a total of ten passes. This procedure produced an inoculum containing elementary bodies without cell debris. Antibiotic-free medium was added to triplicate inoculated McCoy cells as control. Monolayers were stained with a fluorescent antibody reagent, (Ortho Diagnostics). Layers were then examined microscopically, and the number of normal inclusions, if any, was determined.

### RESULTS

Five clinically recovered isolates of *C. trachomatis* were tested against sulbactam, ampicillin/sulbactam, doxycycline, and erythromycin. Six isolates were utilized in the clindamycin group. Good activity against *C. trachomatis* was demonstrated by all antibiotics at normally achievable serum levels with the exception of sulbactam. Percent inhibitions at various antibiotic concentrations are listed in Table I. Minimal concentrations to inhibit 90% growth ($MIC_{90}$) were <0.25 μg/mL for clindamycin, <2.0 μg/mL for erythromycin, <4.0 μg/mL for doxycycline, <8.0 μg/mL for ampicillin/sulbactam, and >64 μg/mL for sulbactam alone.

Normal inclusions were noted following antibiotic exposure to sulbactam, clindamycin, doxycycline, and erythromycin as well as the control groups, and were regular in shape and stained uniformly in brilliant green (Fig. 1). Abnormal inclusions were smaller, misshaped, appeared hollow, and stained faintly (Fig. 2), and were noted only following exposure to ampicillin/sulbactam.

Maximum inhibition occurred after the antibiotic-treated pass (first pass), and regrowth of inclusions to pretreated levels occurred with ampicillin/sulbactam, sulbactam alone, and clindamycin. Regrowth to control levels occurred after a mean of six passes. No regrowth of *C. trachomatis* occurred with doxycycline or erythromycin at any for the ten passes performed following exposure to the initial concentrations of antibiotic.

### DISCUSSION

Several antibiotics have demonstrated varying levels of activity against *C. trachomatis*. However, the

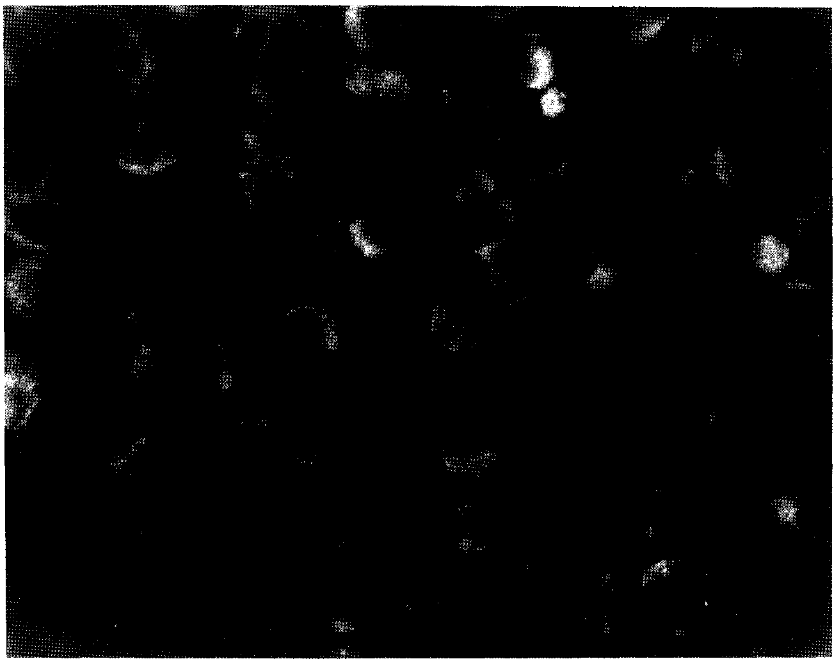

Fig. 1. Normal inclusion. Fluorescent antibody stain, ×400.

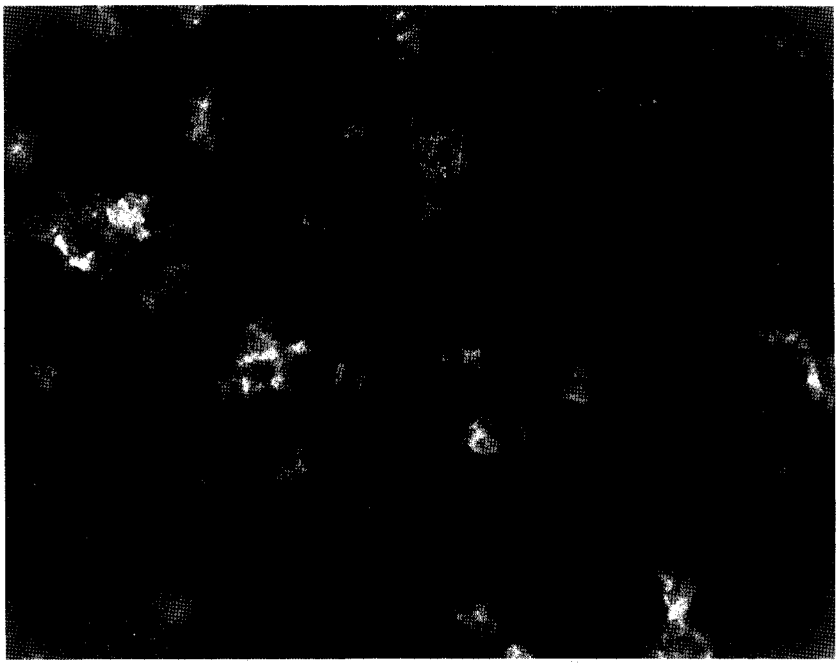

Fig. 2. Abnormal inclusion. Fluorescent antibody stain, ×400.

tetracyclines and erythromycins, as well as their derivatives, have consistently demonstrated excellent in vitro and in vivo activity. Contraindications in pregnant patients, compliance, or tolerance problems have prompted the investigation of other antimicrobial agents. Penicillin class antibiotics are believed to be generally safe in pregnancy and are often better tolerated than erythromycin. Several investigations have demonstrated excellent in vitro and in vivo activity, as mentioned earlier. However, susceptibility testing of *C. trachomatis* to penicillin is complicated by the production of abnormal inclusion bodies and the difficulty of long-term clinical follow-up necessary for correlation of in vivo and in vitro findings.

The abnormal inclusions are readily detectable by FA staining (Figs. 1, 2), and this perhaps should be the method of choice for detecting inclusions during *C. trachomatis* susceptibility testing with the penicillins. However, the use of FA staining and the detection of abnormal inclusions compromises some investigators' definition of MIC and MBCs. Heretofore, the MIC values represented the concentration of antibiotic that inhibited the development of inclusions in McCoy cells. The MBC value represented the concentration of antibiotics that prevented regrowth of inclusions in antibiotic-free McCoy cells. Other investigators have utilized a variety of means to compare the activity of penicillin and other antibiotics. Bowie utilized MIC, MBC, and ≥99% decrease in inclusion formation.[4] Martin et al. utilized percentage inhibition at various antibiotic concentrations.[5] However, until the viability and importance of abnormal inclusions are better understood, great variability in the reporting of anti-chlamydial activity will probably continue.

In this investigation, ampicillin/sulbactam, clindamycin, doxycycline, and erythromycin demonstrated good in vitro activity at concentrations readily achievable in human serum. Only doxycycline and erythromycin eliminated all inclusions consistently, with no regrowth of inclusions, despite ten passes. While the penicillins failed to eradicate inclusion formation entirely, their activity was comparable to clindamycin when examined at the extremely high inoculum utilized in these tests. Therefore, in patients being treated for pelvic or other infections with ampicillin/sulbactam or clindamycin, additional coverage of chlamydial infections with erythromycin and doxycycline may not be necessary. Perhaps reculturing of the patient can demonstrate eradication of the chlamydia, and save the patient the additional expense and complications of these antibiotics.

The regrowth of the clindamycin-treated cells can be explained by the incomplete eradication of *C. trachomatis* at lower doses of clindamycin with subsequent regrowth of few, but viable, remaining inclusions. However, one sample demonstrated regrowth after four passes in which no inclusions were noted after antibiotic exposure, perhaps signifying elementary bodies in the absence of formed inclusions. Thus, the cause of inclusion regrowth with ampicillin/sulbactam may not necessarily be due to the abnormal inclusions, but to a similar regrowth of low numbers of inclusions or individual elementary bodies. Regardless of the etiology of regrowth of inclusions after subsequent antibiotic-free passes in vitro, it is difficult to utilize these results to predict in vivo efficacy.

Sulbactam, by itself, demonstrated poor activity against *C. trachomatis,* similar to previously published reports with other β-lactamase inhibitors.[4] However, the combination ampicillin/sulbactam demonstrated good activity, with a reduction in inclusion counts similar to clindamycin at achievable serum levels. Clindamycin has been recommended for the treatment of *C. trachomatis* by the Centers for Disease Control,[18] although it is not their primary agent due to doxycycline's excellent activity. Penicillin's activity in clinical trials is encouraging, if limited. In vivo, both antibiotics' activities may be augmented by host defenses that combine with the antibiotic's activity to eradicate the infection. However, because of the regrowth of inclusions after initial inhibition, adequate clinical trials need to be completed. Emphasis on long-term follow-up is critical in order to assess the possible regrowth of previously inhibited, but not eradicated inclusions.

The high inoculum utilized in this investigation is artificially achieved, and probably not similar to actual clinical infections. However, it confirms the excellent activity of the tetracyclines and erythromycins, while still highlighting good activity for those antibiotics that may be bacteriostatic, if not bacteriocidal.

Thus, in summary, in vitro susceptibility testing of *C. trachomatis* confirmed the excellent activity of doxycycline and erythromycin. Clindamycin and ampicillin/sulbactam demonstrated good activity, with greater than 99% reduction of inclusion counts at achievable serum concentrations. Sulbactam demonstrated limited activity, consistent with other β-lactamase inhibitors. While clindamycin and ampicillin/sulbactam cannot at this time be recommended as the primary treatment for chlamydial infections, those patients receiving these antibiotics for other indications, in which a chlamydial culture is reported positive, may not necessarily need additional, automatic treatment with doxycycline or erythromycin. However, documentation of eradication of *Chlamydia* by repeat tissue culture is recommended.

### REFERENCES

1. Schacter J, Grossman M: Chlamydia infections. Annu Rev Med 32:45–61, 1981.
2. Kuo CC, Wang SP, Grayston JT: Antimicrobial activity of several antibiotics and sulfonamide against *Chlamydia trachomatis* organisms in cell culture. Antibicrob Agents Chemother 12:80–83, 1977.
3. Walsh M, Kappus EW, Quinn TC: In vitro evaluation of CP-62,933 erythromycin, clindamycin and tetracycline against *Chlamydia trachomatis*. Antibicrob Agents Chemother 31:811–812, 1987.
4. Bowie WR: In vitro activity of the clavulanic acid, amoxicillin and ticarcillin against *Chlamydia trachomatis*. Antibicrob Agents Chemother 29:713–715, 1986.
5. Martin DH, Pastorek JG, Faro S: In vitro and in vivo activity of parenterally administered β-lactam antibiotics against *Chlamydia trachomatis*. Sex Transm Dis 13:81–87, 1986.
6. Noguera X, Ferrer M, Ortola E, Lopez-Marin L: Evaluation of doxycycline in the treatment of urethritis and cervicitis caused by *Chlamydia trachomatis*. Clin Ther 9(Suppl A):33–37, 1986.
7. Lee CK, Bowie WR, Alexander ER: In vitro assays of the efficacy of antimicrobial agents in controlling *Chlamydia trachomatis* propagation. Antimicrob Agents Chemother 13:441–445, 1978.
8. Bowie WR: Lack of in vitro activity of cefoxitin, cefamandole, cefuroxime and piperacillin against *Chlamydia trachomatis*. Antibicrob Agents Chemother 21:339–340, 1982.
9. Bowie WR, Alexander ER, Holmes KK: Eradication of *Chlamydia trachomatis* from the urethras of men with nongonococcal urethritis by treatment with amoxicillin. Sex Transm Dis 8:79–81, 1981.
10. Bowie WR, Manzon LM, Borrie-Hume CJ, Fawcett A, Jones PK: Efficacy of treatment regimens for lower urogenital *Chlamydia trachomatis* infection in women. Am J Obstet Gynecol 142:125–129, 1982.
11. Mourad A, Sweet RL, Sugg N, Schacter J: Relative resistance to erythromycin in *Chlamydia trachomatis*. Antimicrob Agents Chemother 18:696–698, 1980.
12. Bailey JMG, Heppleston C, Richmond SJ: Comparison of the in vitro activities of ofloxacin and tetracycline against *Chlamydia trachomatis*. Antibicrob Agents Chemother 26:13–16, 1984.
13. Hammerschlag MR, Gleyzer A: In vitro activity of a group of broad spectrum cephalosporins and other β-lactam antibiotics against *Chlamydia trachomatis*. Antibicrob Agents Chemother 23:493–494, 1983.
14. Hobson D, Lee N, Bushell AC, Withana N: Activity of β-lactam antibiotics against *Chlamydia trachomatis* in McCoy cell cultures In Mardh PA, Holmes KK, Oriel JD, Piot P, Schachter J (eds): Chlamydial Infections. Amsterdam: Elsevier Biomedical Press, p 249, 1982.
15. Ridgway GL, Owen JM, Oriel JD: The antimicrobial susceptibility of *Chlamydia trachomatis* in cell culture. Br J Vener Dis 54:103–106, 1978.
16. Segreti J, Kessler HA, Kapell KS, Trenholem GM: In vitro activity of A-56268 (TE-031) and four other antimicrobial agents against *Chlamydia trachomatis*. Antibicrob Agents Chemother 31:100–101, 1987.
17 Martens MG, Faro S: β-lactam antibiotics and *Chlamydia trachomatis*. Adv Ther 5:113–118, 1988.
18. Centers for Disease Control: STD treatment guidelines. MMWR 34:1–4S, 1985.
